# Divergent marine anaerobic ciliates harbor closely related *Methanocorpusculum* endosymbionts

**DOI:** 10.1093/ismejo/wrae125

**Published:** 2024-07-10

**Authors:** Anna Schrecengost, Johana Rotterová, Kateřina Poláková, Ivan Čepička, Roxanne A Beinart

**Affiliations:** University of Rhode Island, Graduate School of Oceanography, 215 South Ferry Rd, Narragansett, RI 02882, United States; University of Rhode Island, Graduate School of Oceanography, 215 South Ferry Rd, Narragansett, RI 02882, United States; Department of Marine Sciences, University of Puerto Rico Mayagüez, Mayagüez, United States; Department of Zoology, Faculty of Science, Charles University, Viničná 7, 128 00 Prague 2, Czech Republic; Department of Zoology, Faculty of Science, Charles University, Viničná 7, 128 00 Prague 2, Czech Republic; University of Rhode Island, Graduate School of Oceanography, 215 South Ferry Rd, Narragansett, RI 02882, United States

**Keywords:** anaerobic protists, anoxic sediments, archaea, methanogens, Metopida, Plagiopylea, symbiosis, syntrophy

## Abstract

Ciliates are a diverse group of protists known for their ability to establish various partnerships and thrive in a wide variety of oxygen-depleted environments. Most anaerobic ciliates harbor methanogens, one of the few known archaea living intracellularly. These methanogens increase the metabolic efficiency of host fermentation via syntrophic use of host end-product in methanogenesis. Despite the ubiquity of these symbioses in anoxic habitats, patterns of symbiont specificity and fidelity are not well known. We surveyed two unrelated, commonly found groups of anaerobic ciliates, the Plagiopylea and Metopida, isolated from anoxic marine sediments. We sequenced host 18S rRNA and symbiont 16S rRNA marker genes as well as the symbiont internal transcribed spacer region from our cultured ciliates to identify hosts and their associated methanogenic symbionts. We found that marine ciliates from both of these co-occurring, divergent groups harbor closely related yet distinct intracellular archaea within the *Methanocorpusculum* genus. The symbionts appear to be stable at the host species level, but at higher taxonomic levels, there is evidence that symbiont replacements have occurred. Gaining insight into this unique association will deepen our understanding of the complex transmission modes of marine microbial symbionts, and the mutualistic microbial interactions occurring across domains of life.

Microbial eukaryotes, or protists, are among the most common and abundant organisms on the planet, representing most of the diversity of eukaryotic life. Many protist lineages have evolved to live without oxygen, and these anaerobic protists frequently form symbioses with bacteria and archaea [1, 2]. For those with a fermentative metabolism, symbioses with hydrogen-scavengers may have facilitated transitions to an anaerobic lifestyle [1, 3]. Many anaerobic protists host methanogens in their cytoplasm adjacent to host mitochondria-related organelles (MROs). These MROs generate energy for the host via fermentation, ultimately producing H_2_ which the symbionts utilize as a substrate for methanogenesis. This syntrophic transfer is thought to reduce intracellular hydrogen tension, increasing host metabolic efficiency and enabling relatively large, highly active grazers to thrive in anoxia [4–6]. Despite their ubiquity and diversity in oxygen-depleted habitats ranging from marine sediments to gastrointestinal tracts, basic information about these partnerships, such as the factors that influence which bacterial or archaeal lineages they partner with, is poorly known.

Ciliates are particularly well-suited for life without oxygen: virtually all ciliate lineages include representatives adapted to full or partial anaerobic lifestyles, and most of the recent research on free-living anaerobic protists centers around ciliates [3, 4, 7–9]. However, little is known about symbiont diversity and specificity within and among host ciliate lineages, especially those from marine environments. Most studies have focused on a few host species isolated from a handful of geographically isolated, freshwater habitats [3]. Even so, there is evidence that these associations are stable and may be vertically transmitted: the symbionts divide synchronously in one host species (*Plagiopyla frontata*) [10], and endosymbiotic methanogens persist in host resting cysts [11]. Additionally, identical symbiont species have been detected in a single ciliate species isolated from geographically distant locations [7, 12]. In contrast, the close phylogenetic relationships observed between methanogenic endosymbionts and their free-living relatives suggest that endosymbiont replacements have occurred during their evolution [8, 9, 11]. Studies to date have shown no evidence of co-diversification which would be typical of vertically transmitted symbionts [11, 13]. At the same time, interpretation of these studies is confounded by many variables, including a diversity of geographic locations, habitats, and host phylogeny.

Here, we surveyed the symbionts of two obligately anaerobic genera, *Metopus* and *Plagiopyla*, which are common in oxygen-depleted marine habitats. These two genera are phylogenetically divergent, belonging to different ciliate classes, [14] but co-occur in the shallow, intertidal sediments we sampled here (See online supplementary material for a colour version of Table S1 and Fig. S1). Therefore, we minimized geographical and environmental differences, allowing us to focus on differences in symbiont specificity both within and among these two groups. We identified the methanogenic symbionts from 31 different *Plagiopyla* and 14 different *Metopus* isolates, representing 12 species-level ciliate lineages (Figs 1 and 2, See online supplementary material for a colour version of Fig. S1; Supplemental Information) [15]. We found that, despite their large phylogenetic diversity and geographic range, all of these ciliates harbor closely-related *Methanocorpusculum* symbionts whose 16S rRNA gene sequences are 98%–100% identical (mean 99% ∓ 0.54 s.d.), with one exception (a *Methanolacinia* symbiont*,*Fig. 1D). These symbionts are also closely related to free-living *Methanocorpusculum* species (Fig. 1D, Table S4). *Methanocorpusculum* has primarily been recorded in wastewater environments and as an endobiont of animals and anaerobic ciliates [7, 12, 16, 17]. Transmission electron microscopy (TEM) revealed that the *Methanocorpusculum* symbionts from representatives of both host groups are highly integrated with the host cell, located in between host MROs (Fig. 1). These findings are similar to those from studies of *Methanocorpusculum* in other ciliates, suggesting a tight integration with the host on both a structural [13] and genomic [7] level is a feature which distinguishes this genus from other symbiotic methanogens.

**Figure 1:**
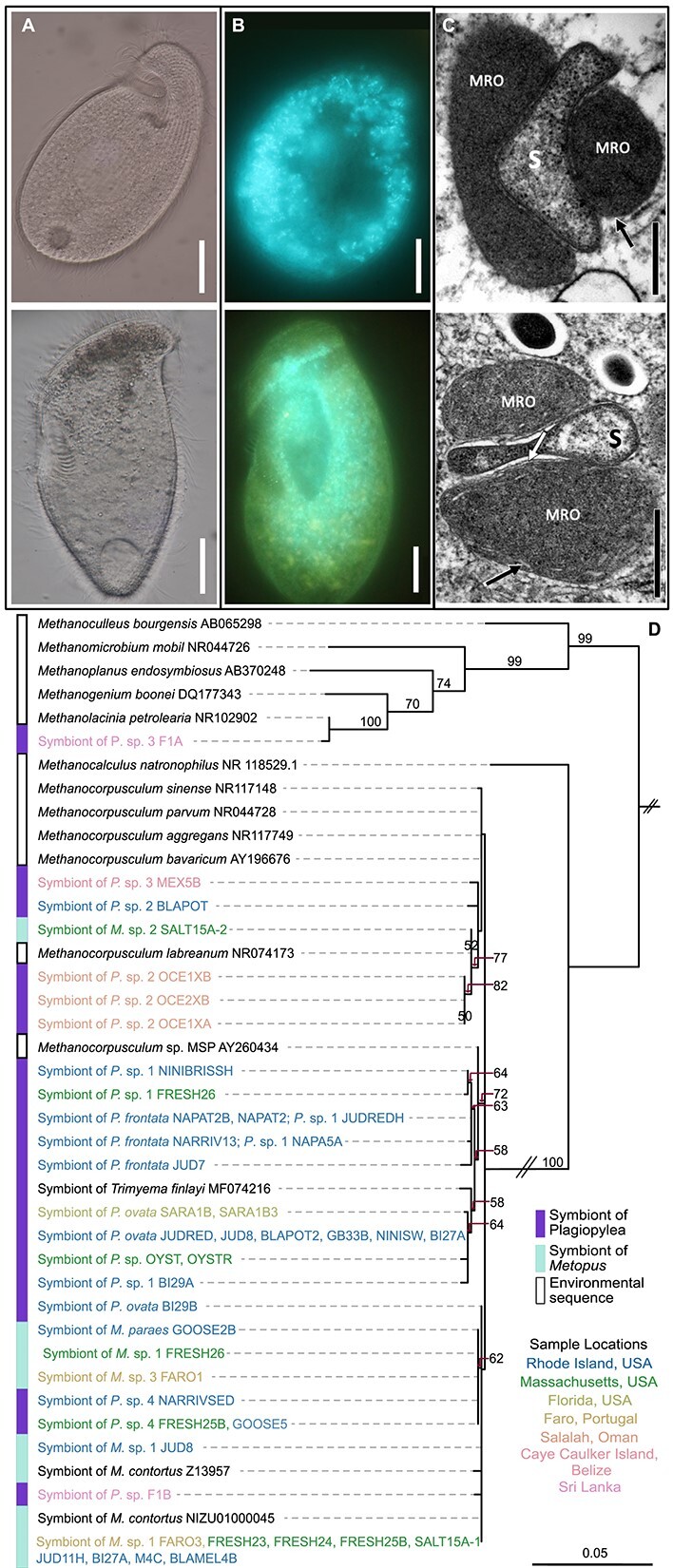
(A) Representative light micrographs of host *Plagiopyla* (NAPAT2, top) and *Metopus* (BI27A, bottom) cells. (B) Representative micrographs showing autofluorescence of methanogenic symbionts within host *Plagiopyla* (OYST, top) and *Metopus* (FRESH26, bottom) cells. (C) Transmission electron microscopy (TEM) depicting the ultrastructure of methanogenic symbionts within host *Plagiopyla* (FRESH26, top) and *Metopus* (BLAMEL4B, bottom). (D) Phylogenetic tree of 16S rRNA gene sequences of the methanogenic symbionts obtained in this study along with published sequences from cultured representatives as well as environmental sequences, and other published ciliate symbiont sequences. Symbiont sequences recovered in this study are colored by sample site (described in Fig. S1, Table S1). Double dashes on branches indicate that branch was shorted to 25% of the original length. Bootstrapping was performed 1000 times and only values above 50 are shown. In (A)-(B), scale bar = 20 µm. In (C), scale bar = 500 nm; MRO = mitochondrion related organelles; S = symbiont; black arrow = remnants of mitochondrial cristae; white arrow = mitochondrial double membrane.

**Figure 2:**
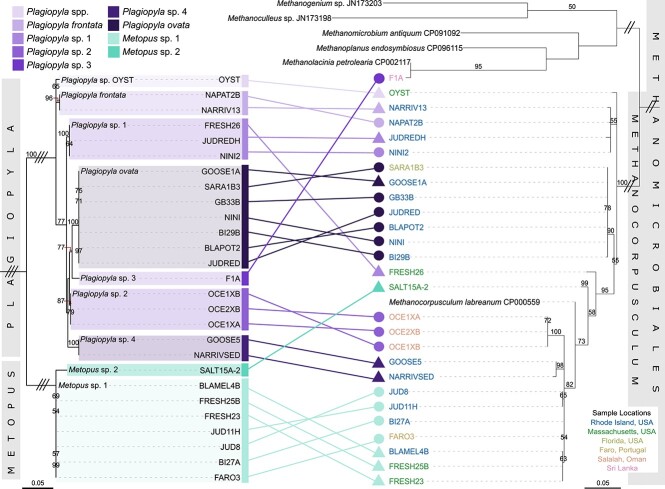
Paired host (left) and symbiont (right) phylogenetic trees. Host trees are based on the 18S rRNA gene sequences of the ciliates isolated in this study, and symbiont trees are based on the symbiont ITS region. Host and symbiont sequences are connected by lines, and nodes are colored by host species group. Lines connecting nodes are colored by sample site (described in Fig. S1, Table S1). Triangle-shaped nodes represent strains which were cultured in brackish media, and circular nodes represent strains which were cultured in fully marine media. Triple and double dashes on branches indicate that branches were shortened to 10% and 25% of the original length, respectively. Bootstrapping was performed 1000 times and only values above 50 are shown.

To provide higher resolution for distinguishing these symbionts (Fig. 1), in a subset of ciliates, we sequenced the symbiont 16S/23S internal transcribed spacer region (ITS) in addition to the 16S rRNA gene. This is commonly used to detect strain-level differences among bacteria and archaea, including methanogens [18] (Fig. 2). We found that *Plagiopyla* and *Metopus* host closely related yet distinct *Methanocorpusculum* endosymbionts, even when they co-occurred at the same location or in the same culture (e.g. FRESH25B, FRESH26, and JUD8; Fig. 1, Fig. 2). Additionally, symbionts cluster according to host species even when they originate from very distant locations or disparate habitats. For example, *Metopus* sp. 1 includes representatives isolated from the USA and in Portugal, and all its symbiont ITS sequences are identical (Fig. 2). Symbionts of all *Plagiopyla ovata* populations are identical as well and include isolates from salt pond sediments in New England as well as mangrove sediments in Florida. Within host species, we sometimes see strain-level differentiation by geography: symbionts of *Metopus* sp. 1 isolates from sites 75 km apart formed site-specific clades despite hosts being virtually identical at the 18S rRNA gene level (FRESH and JUD, Fig. 2). Even so, host-symbiont phylogenies are not congruent and, in some cases, *Metopus* and *Plagiopyla* symbionts are more related to each other than to symbionts from other species within their respective genera (Fig. 2).

Here, we found that two, co-occurring, divergent marine ciliate lineages harbor very closely related *Methanocorpusculum* endosymbionts. These symbionts appear to be stable at the host species level, most likely through vertical transmission via synchronous division [10], but at higher taxonomic levels, there is evidence that symbiont swaps/replacements have occurred. Mixed-mode transmission is common in cross-domain symbioses, especially those in aquatic environments [19]. Particularly for marine symbioses, it has been found that mixed-mode transmission can result in long-term evolutionary stability without extreme symbiont genome erosion via a combination of guaranteed transmission and symbiont replacement, while also allowing the host to associate with a locally adapted symbiont [20]. Although it is ubiquitous in anoxia, this association is unique – the methanogenic symbionts of ciliates and other protists represent some of the few known intracellular archaea. Understanding the evolutionary dynamics of this association will provide insight into the complex mechanisms which shape the mixed-mode transmission strategies of cross-domain microbial symbioses. Future work using comparative and population genomics will be necessary to better help us understand how mixed-mode transmission shapes the ecology and evolution of these common yet complex partnerships.

## Supplementary Material

FigureS1_wrae125

FigureS2_wrae125

Table_S1_wrae125

Table_S2_wrae125

Table_S3_wrae125

Table_S4_wrae125

Table_S5_wrae125

Table_S6_wrae125

Supplementary_information_wrae125

## Data Availability

Host 18S rRNA gene sequences for *Plagiopyla frontata* and *Plagiopyla ovata* are available on GenBank (accession numbers OP186382-OP186395, previously published). The remaining sequences (host 18S and symbiont 16S rRNA genes and ITS region sequences) are available on GenBank (accession numbers PP213518-PP213548 and PP835390-PP835394; PP438670-PP438713, and PP215653-PP215679 and PP229184, respectively.
