# Supplementary figures and images for: Divergent marine anaerobic ciliates harbor closely related Methanocorpusculum endosymbionts
Source: ISME J. 2024 Jul 10;18(1):wrae125. doi: 10.1093/ismejo/wrae125 (PMC11253715; doi:10.1093/ismejo/wrae125)

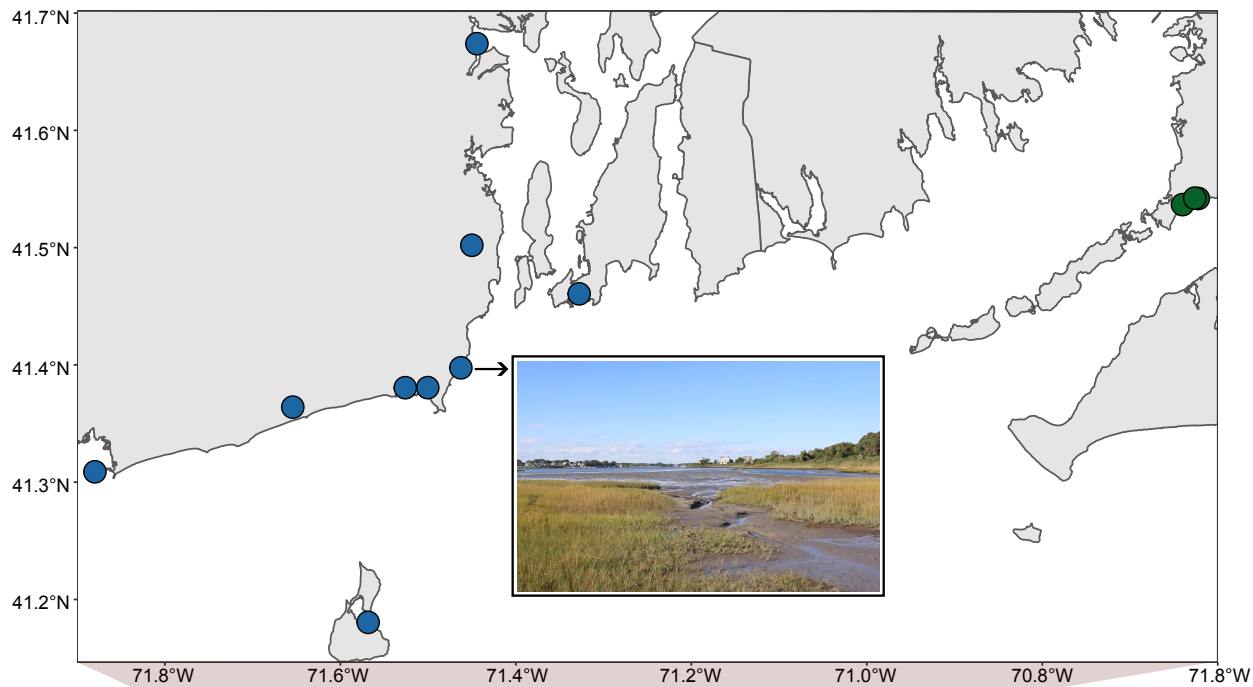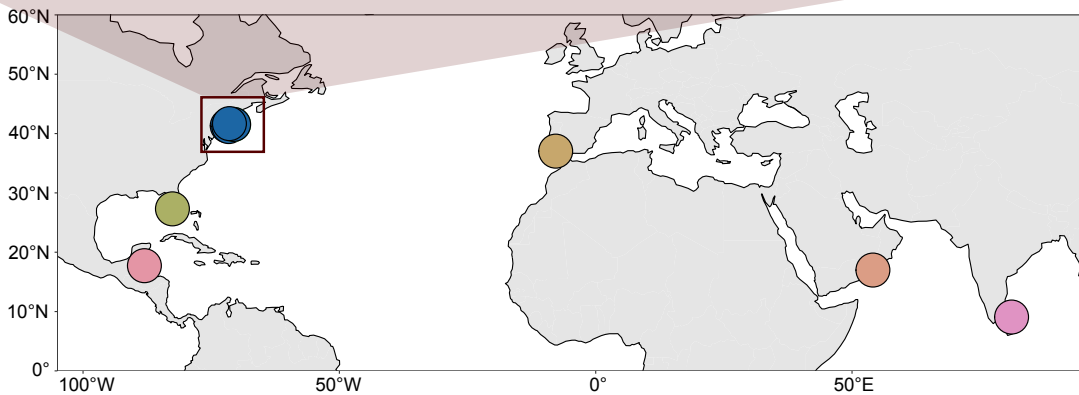

Supplement: FigureS1_wrae125 [file figures1_wrae125.pdf]

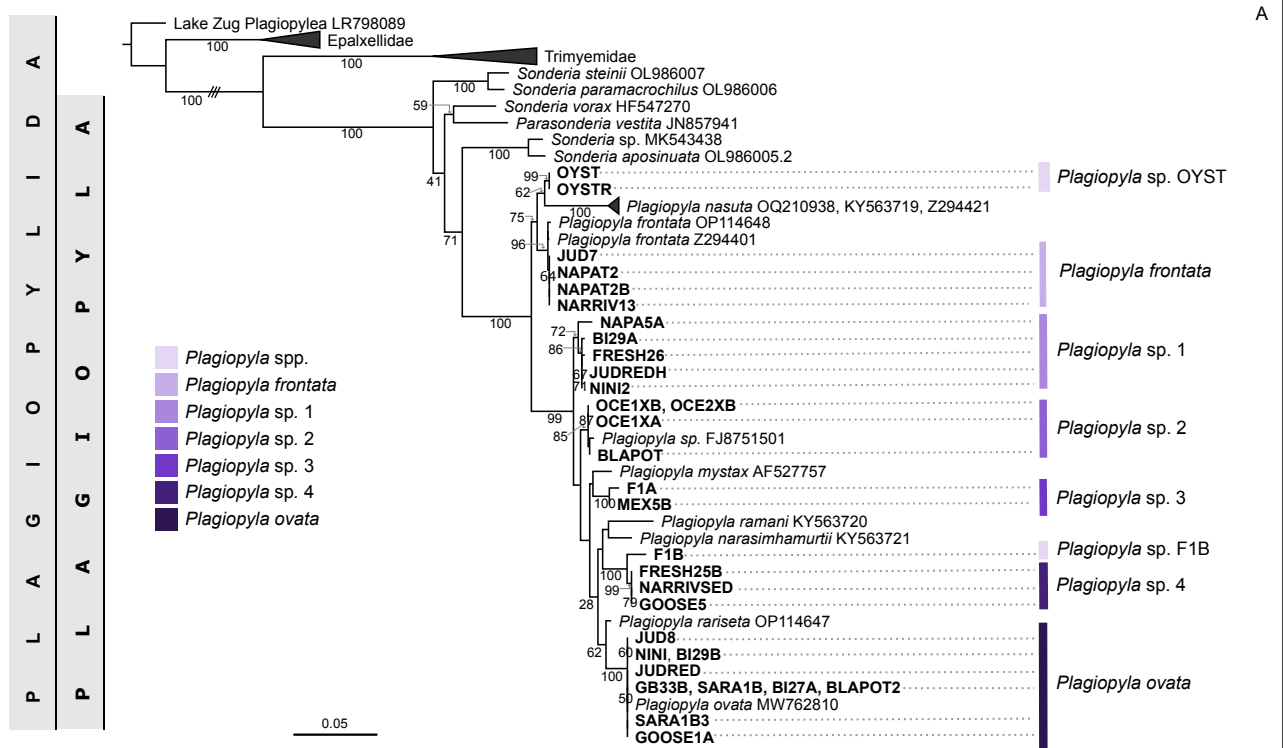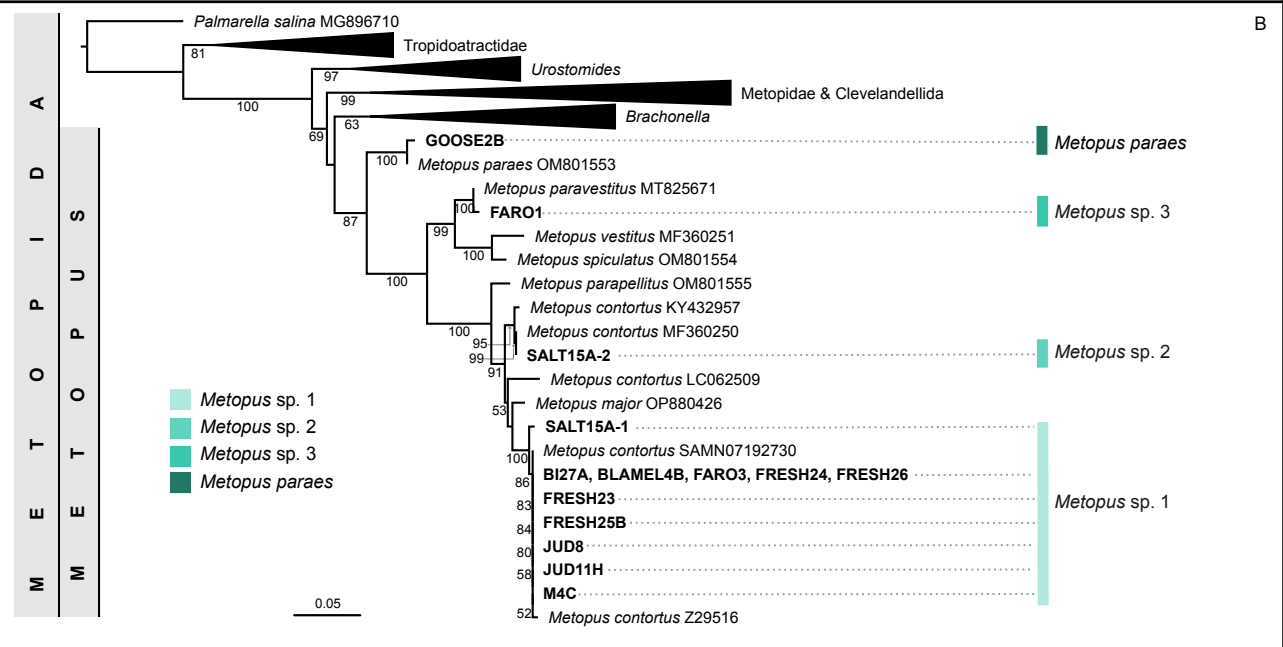

Supplement: FigureS2_wrae125 [file figures2_wrae125.pdf]
